# Understanding the Emotion Coping Strategies During Public Emergencies – From the Perspective of Psychological Distance

**DOI:** 10.3389/fpsyg.2021.699180

**Published:** 2021-11-05

**Authors:** Yan Sun, Yang Li, Yong Wang, Fangmin Li

**Affiliations:** ^1^Key Laboratory of Behavioral Science, Institute of Psychology, Chinese Academy of Sciences, Beijing, China; ^2^Department of Psychology, University of Chinese Academy of Sciences, Beijing, China; ^3^Business School, Beijing Technology and Business University, Beijing, China; ^4^School of Psychological and Cognitive Sciences, Peking University, Beijing, China

**Keywords:** psychological distance, negative emotion, COVID-19 outbreak, copying strategies, self-construal

## Abstract

Research has investigated behavioral coping strategies for the negative emotions that public emergencies elicit. Accordingly, our current research explored how people coped with negative emotions in response to the coronavirus disease (COVID-19) outbreak, from a cognitive perspective. Building on the theory of psychological distance and self-construal, we proposed that people who experienced fear, sadness and anxiety responded with independent-self construal, focusing on information that related to themselves and the novel virus (independent information). On the other hand, people who experienced fear, sadness and anger responded with interdependent-self construal, focusing on information that pertained to “us”, the virus and nature (interdependent information). We collected data from 1,142 participants at both the initial peak of the outbreak and when its spread had subsided. Based on this longitudinal data, we examined the effectiveness of these strategies, and our findings suggested that independent information was effective in decreasing fear and anxiety, while interdependent information effectively mitigated sadness. The findings could help researchers, practitioners, governments, and organizations to implement appropriate information strategies to regulate individuals’ negative emotions during and after the COVID-19 pandemic.

## Introduction

Scholars have focused on the negative emotions that disasters elicit (Folkman, 2008; Yiend, 2010), since the negative emotions may trigger mental health issues in the long-term (Kiecolt-Glaser et al., 2002). Existing literature has confirmed that people have suffered increasing negative emotions during the novel coronavirus (COVID-19) pandemic (Elhai et al., 2020; Li W. et al., 2020), and were still exploring ways to cope with these emotions. Unfortunately, the authorities implemented lockdown measures, requiring people to remain at home, which obviously limited behavioral coping strategies. Thus, we could not reliably measure the efficacy of these coping strategies. Our research addresses these difficulties by examining the effectiveness of cognitive coping strategies, using longitudinal data.

People tend to regulate their emotions when facing adverse situations (Waugh, 2020), but the approaches employed to cope with negative emotions vary accordingly (Piff et al., 2012). Researchers have investigated how people recovered from social emergencies, such as bushfires (Eriksen, 2019), hurricanes (Morrice, 2012), and Severe Acute Respiratory Syndrome (SARS) (Maunder et al., 2003). However, the COVID-19 pandemic is different since, in many countries, authorities have required people to stay at home for extended periods. The lockdown has proven to be effective in slowing the spread of the virus (Kupferschmidt, 2020), but the associated psychological cost cannot be ignored (Rubin and Wessely, 2020). Hence, it is imperative to identify ways to cope with the negative emotions that the pandemic has prompted.

According to the transactional model of coping (Lazarus and Folkman, 1984), people tend to employ various approaches to address negative emotions (Folkman, 2008). In general, there are two ways of coping—the direct way to solve the problems that elicit negative emotions (problem-focused coping), or the indirect way that people use to distance themselves from negative emotions (emotion-focused coping). These coping strategies depend on the domain of threat; a mortal threat, like the COVID-19 pandemic, would more likely activate problem-focused coping (Han et al., 2015). With this approach, people would actively search for solutions to the current situation.

The coping strategies that people use to regulate their emotions could be either behavioral or cognitive efforts (Gross, 1998; Gross and John, 2003). Due to the limitation and inflexibility of behavioral approaches during the outbreak of COVID-19, our research discusses coping strategies at the cognitive level. Specifically, we examine the information that individuals associated with different negative emotions. Based on the connection between negative emotions and information searching, we propose that people with certain negative emotions will only pay attention to relevant information (Kustubayeva et al., 2012; Tamir, 2016).

We classified two kinds of information based on the theory of self-construal (Markus and Kitayama, 1991). Since individuals focus on themselves either independently or interdependently, we proposed that they would consider themselves as “me” or as “a part of the human collective (us)” during the pandemic. People who understood the situation from the perspective of the independent-self focused more on the threats to individuals and attempted to solve the “me” problems. Hence, they searched for updated information that related to the status of the virus and the ways they could protect themselves. In this paper, we defined these kinds of information as “independent information.” Alternatively, people who understood the situation in terms of the interdependent-self paid more attention to the threats to a collective “us.” Thus, they intended to solve the problems of “the human collective,” to decrease the possibility of such disasters in the future. Therefore, they considered the relationship between humans and wildlife, or the harmony of nature. In this paper, we named these kinds of information as “interdependent information.” Markus and Kitayama (1991) stated that emotions that people were experiencing would foster either independent-self or interdependent-self construal.

Taken together, we examined five negative emotions (fear, sadness, anxiety, anger, and disgust) that the outbreak of COVID-19 visibly induced (Bao et al., 2020; Li W. et al., 2020; Limcaoco et al., 2020). We proposed that people searched for independent and interdependent information to regulate negative emotions, and the effectiveness of the coping strategies employed would vary according to the emotion. We conducted a longitudinal study to compare emotional intensity and the relevant coping strategies at different times. We collected the first round of data at the initial peak of the number of infections in China (T_1_). We then obtained the second round of data when there were no new reported cases of infections (T_2_).

To the best of our knowledge, this study is the first to examine the coping strategies that address negative emotions during the COVID-19 pandemic, based on longitudinal data. This is meaningful for the following reasons. First, this study discusses coping strategies from the cognitive rather than behavioral perspective. Changes in social behaviors would bring immediate effect, for example, social connection could promptly decrease anxiety caused by social distancing (Williams et al., 2018). However, it remains imprudent for individuals from severely infected areas to return immediately to normal physical interactions. Therefore, cognitive coping strategies were more influential during the pandemic. Second, our novel study distinguishes problem-focused coping, building on the theory of psychological distance and self-construal. We classify independent information (related to the status of virus) and interdependent information (related to relationship between human beings and nature). This is significant for future research to understand how people think and what kinds of information they need during major public emergencies. Third, we examine the effectiveness of the two coping strategies in reducing negative emotions based on the data from T_1_ to T_2_. The findings may provide solid support for future studies on the mitigation of negative emotions during major public emergencies.

Extensive literature has confirmed that public emergencies can induce negative emotions (Maunder et al., 2003; Morrice, 2012; Eriksen, 2019), while the uncertainty and unpredictability of public health crises would prompt such responses more intensely (Slovic, 1987). However, countries, communities, and individuals all need time to recover from such crises. For individuals, one of the critical processes during recovery is to regulate the emotions prompted by negative events (Eriksen, 2019). Negative emotion regulation is a series of coping strategies that individuals use to alleviate their emotional states (Gross, 1998), and it varies with individual characteristics and situations (Dunkley et al., 2003; Piff et al., 2012; Bonanno and Burton, 2013). Scholars have explored abundant coping strategies to deal with negative emotions. These include the reversal of negative emotions to positive emotions (Folkman and Moskowitz, 2000; Tugade and Fredrickson, 2004), forgetting about negative emotions (e.g., diverting attention to other things) (Shimazu and Schaufeli, 2007; Cameron and Payne, 2011), reappraising the meaning of negative events (Park and Folkman, 1997; Knoll et al., 2009), and finding solutions to resolve current problems (Witte and Allen, 2000).

In summary, there are two kinds of coping strategies: problem-focused and emotion-focused coping (Lazarus and Folkman, 1984; Folkman, 2008, 2009) and both of them involve behavioral or cognitive efforts to alleviate negative emotions (Duhachek, 2008). At the cognitive level, emotional regulation could be a goal-oriented process (Tamir, 2016), and people tend to focus on different information that corresponds to their emotions. Peters et al. (2006) stated that individuals heeded emotional information rather than neutral information after the induction of a particular emotion. However, attention would shift if provided with information that alleviated negative emotions (Vogt et al., 2011; Vogt and De Houwer, 2014). A similar pattern emerged during the COVID-19 outbreak; individuals increasingly consumed negative information after the prompting of negative emotions (Van Bavel et al., 2020). In terms of information depth, previous literature suggested that various emotions would induce different depths of information focus (Wang et al., 2017), which in turn bring a distant perspective (Bruehlman-Senecal and Ayduk, 2015).

When discussing emotion regulation from the theory of psychological distance and self-construal, the emotions could reinforce an independent or interdependent construal of the self (Markus and Kitayama, 1991). Specifically, the ways (construal) in which people consider their situations vary according to their emotions and affects their information focus. Individuals who recognized their particular vulnerability during the COVID-19 outbreak would focus more on information that closely related to status of the virus and the associated protective measures. These kinds of information could help solve the present problems of “me” and our current research defined it as “independent information.” In contrast, when individuals recognized themselves as part of a human collective, they would focus more on the relationship between humans and wildlife, or the harmonious development of nature. Such information would be helpful to solve the future problems of “us,” decreasing the possibility of future outbreaks. We defined this as “interdependent information.” Accordingly, we can reasonably assume that independent information can elucidate some perspective for the short-term, while interdependent information can do so for the long-term. However, few studies have connected information focus with the approaches that help cope with negative emotions, and there is a lack of evidence to demonstrate the link between cognitive coping strategies and relevant emotions.

To date, some scholars have studied the negative emotions elicited by the COVID-19 pandemic, such as fear, anxiety, depression, and anger (Bao et al., 2020; Li W. et al., 2020; Limcaoco et al., 2020), but the majority of these studies focused either on one emotion or regarded negative emotions as a whole rather than discussing them separately. Psychologists have emphasized the necessity of distinguishing negative emotions, stating that different emotions prime distinct goals for decision-makers (Raghunathan and Pham, 1999), and highlighted the importance of matching the regulation strategies with specific emotions (Labroo and Rucker, 2010). Grounded on previous research, our study suggested that individuals used their information focus (thinking) to regulate their negative emotions during the COVID-19 outbreak. However, the types of negative emotions (fear, sadness, anxiety, anger, and disgust) prompt distinct construal of thinking, which in turn work differently for each negative emotion. We believe that the causes of each emotion could explain this difference.

Specifically, fear and anxiety are two frequently mentioned emotions since uncertainty is likely to be their source (Raghunathan and Pham, 1999; Griskevicius et al., 2009). The pandemic has elicited these emotions due to the uncertainty resulting from lack of knowledge about the virus (Bao et al., 2020). Hence, fear and anxiety have more internal attributes and experiencing these kinds of emotions prompts the independent-self perspective. Consequently, individuals searched for independent information to regulate their fear and anxiety. As their knowledge increased, their fear and anxiety decreased. In addition, their search for independent information about self-protection prompted them to make further risk-averse choices (Lerner and Keltner, 2001).

Individuals feel sadness often because of their own or others’ misfortunes (Cryder et al., 2008; Labroo and Rucker, 2010). In other words, they have both internal and external attributes, and they relate to themselves and others at the same time. Therefore, experiencing sadness motivates either an independent-self or an interdependent-self view. The independent construal motivates, for example, a search for information about the status of the virus, the number of new infections and cured cases. Conversely, the interdependent construal motivates a search for information such as the relationship between humans and nature. Compared to the information directly related to the virus, interdependent information would work better in reducing sadness because the former cannot change the current facts. The latter, however, changes individuals’ perception of the negative event (McRae et al., 2012) and elicit the hope that humanity could mobilize its resources and actually mitigate future threats. Therefore, focus on interdependent information induces a reappraisal of the pandemic and regard it as motivation to interact with nature more responsibly, eradicating pandemic-induced sadness in the long-term.

Anger and disgust tend to be the result of others’ misbehaviors (Lerner and Keltner, 2001; Lerner et al., 2008). During the outbreak, individuals felt anger and disgust because authorities linked the advent of COVID-19 to the consumption of wildlife (Lu et al., 2020; Zhou et al., 2020). As they deemed this consumption as one of the main routes for transmitting the virus to humans, individuals became aware of the effects of others’ behaviors. However, this interdependent perspective could not decrease their anger or disgust since they could not find solutions to change others’ perceived misbehaviors.

Therefore, we propose that individuals focused on information that related to the status of the virus (independent information focus) to regulate their fear (H_1a_), sadness (H_1b_), and anxiety (H_1c_), while searching for “distant” information related to the relationship between humans and nature (interdependent information focus) to regulate their fear (H_2a_), sadness (H_2b_), anger (H_2c_), and disgust (H_2__*d*_). Meanwhile, we posit that focus on independent information could significantly reduce fear (H_3a_) and anxiety (H_3b_), while interdependent information could prominently decrease sadness (H_4_).

The novelty of our study lies in the distinction of “close” and “distant” coping strategies for five kinds of negative emotions: fear, sadness, anxiety, indignation, and disgust. We are also the first to examine the effectiveness of coping strategies based on longitudinal data during the COVID-19 outbreak. We discuss coping strategies from the cognitive level rather than the behavioral level since behaviors would vary with the situations, countries, policies, cultures, or habits; however, there is little limitation on the development of cognition. Therefore, our findings are flexible, whose implementation would be appropriate in China and other countries.

## Materials and Methods

We conducted a longitudinal survey in China through the professional data collection platform, Credamo, in 2020. We delivered the first round of questionnaires on February 24, 2020 (T_1_), when the number of infected cases peaked in China. We delivered the second round on March 30 (T_2_), when the number of reported new infections had returned to zero. In the first round, we obtained 500 valid samples from Hubei province, the most severely affected area, and 1000 valid samples from other provinces of China. In the second round, we delivered the questionnaires to the same participants and received 1,142 valid responses. To guarantee data consistency, we only used paired samples.

### Participants

Of the participants, 565 were male (49%). All subjects were Chinese, ranging from 18 to 65 in age (*M _age_* = 29), with a range of occupational backgrounds: university students (22%), employees of enterprises (44%), staff of the government and institutions (11%), which included doctors (9%), self-employed individuals (14%), farmers (8%), and other occupations (1%). A total of 73% of participants held a Bachelor’s degree while 7% held a Master’s degree.

### Measures

We measured the negative emotions using five items on a seven-point scale, including fear, sadness, anxiety, anger, and disgust (Cronbach’s α = 0.893). We measured the focus on independent information using two items on a seven-point scale (“amount of time spent on thinking or reading information about the virus, including the number of infected cases, the means of protection and other relevant policies” and “number of times you discuss the virus with others, including the number of infected cases, the means of protection and other relevant policies”) (Cronbach’s α = 0.793). The original measure consisted of three items and we deleted one item that did not relate closely to the concept (“number of times that you share information about virus with others”).

We measured focus on interdependent information using five items on a seven-point scale (“thinking or reading about the relationship between the virus and the consumption of wildlife,” “thinking or reading about the relationship between human beings and nature,” “thinking or reading about the relationship between human beings and wildlife,” “the depth of thinking about the above questions,” and “the time spent on thinking or reading about the above information” (Cronbach’s α = 0.872).

All above three variables have high reliability (Hair, 1998) as their Cronbach’s alpha values were above 0.6 (negative emotion 0.893, focus on independent information 0.793, and focus on interdependent information 0.872). Moreover, the measurement has high convergent validity with all square roots of AVE above 0.8 (negative emotion 0.948, focus on independent information 0.86, and focus on interdependent information 0.947) (Fornell and Larcker, 1981).

## Results

Our study explores two questions: (1) How did people cope with their negative emotions during the COVID-19 outbreak, and (2) how effective was each coping strategy? For the first question, we examined the relationship between negative emotions and these coping strategies. To address the second question, we tested the relationship between the coping strategies and the change in each negative emotion.

### Emotional Intensity

**FIGURE 1:**
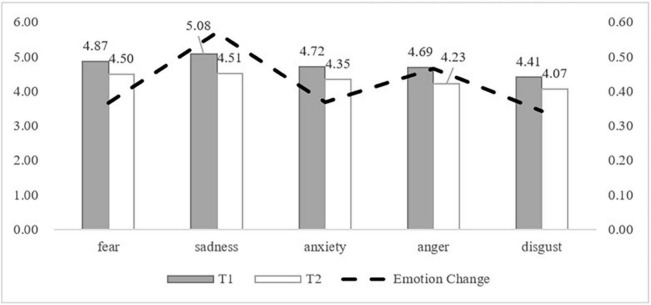
Emotional strength between T1 and T2.

First, we compared the emotional intensity between T_1_ and T_2_ (Figure 1). The emotions of fear (*M_fear__T1_* = 4.87, *M_fear_*_*T2*_ = 4.50, *t*_*fear*_ = 8.005, *p* < 0.001), sadness (*M*_sadness T1_ = 5.08, *M*_sadness T2_ = 4.51, *t*_*sadness*_ = 11.932, *p* < 0.001), anxiety (*M*_*anxiety*__*T1*_ = 4.72, *M*_*anxiety*__*T2*_ = 4.35, *t*_*anxiety*_ = 7.795, *p* < 0.001), anger (*M*_*anger*__*T1*_ = 4.69, *M*_*anger*__*T2*_ = 4.23, *t*_*anger*_ = 9.134, *p* < 0.001) and disgust (*M*_disgust T1_ = 4.41, *M*_disgust T2_ = 4.07, *t*_*disgust*_ = 6.853; *p* < 0.001) all decreased significantly from T_1_ to T_2_. The change in sadness (*M*_*sadness change*_ = 0.57) and anger (*M _ang__er__change_* = 0.46) significantly exceeded that of the other three emotions (*M _fear__change_* = 0.37, *M _anxiety change_* = 0.37, *M _disgust change_* = 0.34).

### Negative Emotions and Coping Strategies

To examine the relationship between the negative emotions and coping strategies at two time periods, we established a Hierarchical Linear Model (HLM) with a random intercept. The follows:


(1) $$\{\begin{array}{c} Y_{i\,j}=\beta_{0\,i\,j}+\beta_{1\,i\,j}\,C_{j}+\beta_{2\,i\,j}\,E_{i\,j}+\varepsilon_{i}\\ \beta_{0\,i\,j}=\gamma_{00\,i}+\alpha_{i\,j}\,P+\mu_{0\,j} \end{array}$$

(2) $$\to Y_{i\,j}=\gamma_{00\,i}+\alpha_{i\,j}\,P+\beta_{1\,i\,j}\,C_{j}+\beta_{2\,i\,j}\,E_{i\,j}+\left(\mu_{0\,j}+\varepsilon_{i}\right)$$

Y_1_ represents the focus on independent information, while Y_2_ represents the focus on independent information. C_*j*_ is a vector of control variables for the j consumer, consisting of his or her demographic information: such as the gender (male is coded 1 and female is coded 0), age and income. P represents the different pandemic periods (0 is the peak of the pandemic, 1 is the point where the number of reported new infections dropped to zero). Eij represents the five different kinds of negative emotions for the consumer j, (*i* = 1 sadness, *i* = 2 disgust, *i* = 3 anger, *i* = 4 anxiety, *i* = 5 fear).

Based on the 1,142 paired samples, we estimated two HLM models to test the influence factors on the cognitive coping strategies. Tables 1, 2 summarized these results.

**TABLE 1:** Effects of emotions on coping strategy of independent information focus.

**Fix effects**	**F**	**Sig**	**Fix coefficients**	**B**	**t**	**sig.**
Corrected Model	36.276	0.000	Intercept	3.885	29.821	0.000
Gender	0.778	0.378	Gender	0.041	0.882	0.378
Age	6.666	0.010	Age	0.009	2.582	0.010
Income	9.777	0.002	Income	0.085	3.127	0.002
Period	73.802	0.000	Period	0.400	8.591	0.000
Sadness	24.276	0.000	Sadness	0.096	4.927	0.000
Disgust	0.155	0.694	Disgust	0.007	0.394	0.694
Anger	0.602	0.438	Anger	0.015	0.776	0.438
Anxiety	7.143	0.008	Anxiety	0.062	2.673	0.008
Fear	5.017	0.025	Fear	0.052	2.240	0.025

			Random effects	B	z	Sig
			Var(period = 0)	0.991	23.792	0.000
			Var(period = 1)	1.407	23.810	0.000

**TABLE 2:** Effects of emotions on coping strategy of interdependent information focus.

**Fix effects**	**F**	**Sig**	**Fix coefficients**	**B**	**t**	**sig.**
Corrected Model	22.662	0.000	Intercept	4.144	38.150	0.000
Gender	0.208	0.649	Gender	–0.018	–0.456	0.649
Age	19.918	0.000	Age	0.013	4.463	0.000
Income	40.752	0.000	Income	0.145	6.384	0.000
Period	0.671	0.413	Period	–0.032	–0.819	0.413
Sadness	18.835	0.000	Sadness	0.071	4.340	0.000
Disgust	0.502	0.479	Disgust	–0.011	–0.709	0.479
Anger	6.526	0.011	Anger	0.041	2.555	0.011
Anxiety	0.234	0.628	Anxiety	0.009	0.484	0.628
Fear	4.169	0.041	Fear	0.040	2.042	0.041

			Random effects	*B*	*z*	Sig
			Var (Period = 0)	0.791	23.824	0.000
			Var (Period = 1)	0.847	23.829	0.000

For the strategy focusing on independent information, the model is significant (*F* = 36.276, *p* < 0.001). Specifically, the coefficients of sadness (*B* = 0.096, *t* = 4.927, *p* < 0.001), anxiety (*B* = 0.062, *t* = 2.673, *p* = 0.008) and fear (*B* = 0.052, *t* = 2.240, *p* = 0.025) are significant, but the coefficients of disgust and anger are insignificant. Hence, H_1a_, H_1b_, and H_1c_ are valid. For the strategy focusing on interdependent information, the HLM model is also well-established (*F* = 22.662, *p* < 0.001). The coefficients of sadness (*B* = 0.071, *t* = 4.430, *p* < 0.001), anger (*B* = 0.041, *t* = 2.555, *p* = 0.011) and fear (*B* = 0.040, *t* = 2.042, *p* = 0.041) are prominent, but the coefficients of disgust and anger are not significant. Hence, H_2a_, H_2b_ and H_2c_ are valid, but H_2d_ is yet unproven.

Therefore, individuals focused on information that related to the status of the virus when they experienced fear, sadness, and anxiety, while considering the relationship between human beings and nature to regulate their fear, sadness, and anger. However, the effectiveness of each coping strategy in reducing negative emotions was still unknown. Accordingly, we examined the effectiveness through checking the change in each emotion during T_1_ and T_2_.

### Coping Strategies and Emotional Change

We constructed a linear regression model (Equation 3) to test the relationship between coping strategies and the change in each negative emotion. Y_3_ represents the change in each negative emotion (T_2_–T_1_). S_1_ represents the consideration of questions pertaining to the status of the virus at the first time point (T_1_). S_2_ represents the consideration questions pertaining to the cause of the virus at the first time point (T_1_). Ci is a vector of control variables consisting of participants’ demographic information: gender, age, income, education background, and place of residence.


(3) $${\text{Y}}_{3}=\beta_{13}\,{\text{S}}_{1}+\beta_{14}\,{\text{S}}_{2}+\beta_{15}\,\text{Ci}$$

We estimated Equation 3 with the change of each negative emotion. The results (see Table 3A–E) indicate that the coefficient of independent information focus was significant when the demographic information was controlled, and if we estimated Equation 3 with the change of fear (β*_13 fear_* = −0.078, *t* = 2.390, *p* = 0.017) and anxiety (β*_13_*_*anxiety*_ = −0.066, *t* = −2.037, *p* = 0.042). Thus, we confirmed H_3a_ and H_3b_. The coefficient of interdependent information became significant based on the change in sadness (β*_14 sadness_* = −0.087, *t* = −2.646, *p* = 0.008). Thus, we validated H_4_.

**TABLE 3:** Effects of coping strategies on the change of each emotion.

**A. Fear**	**Model 1**			**Model 2**		
	**β**	**T**		**β**	**T**	
Gender	0.005	0.171		0.007	0.238	
Age	–0.030	–0.899		–0.026	–0.789	
Income	–0.053	–1.527		–0.047	–1.353	
Education	–0.016	–0.500		–0.020	–0.644	
Place	–0.028	–0.915		–0.026	–0.847	
Independent information focus				–0.078	–2.390	**
Interdependent information focus				0.001	0.042	
*R2*		0.006			0.011	
*F for change in R2*		1.267			3.737	**
*F for Model Fit*		1.104			1.771	*

**B. Sadness**	**Model 1**			**Model 2**		
	**β**	**T**		**β**	**T**	

Gender	–0.035	–1.158		–0.032	–1.066	
Age	–0.068	–2.067	**	–0.058	–1.737	*
Income	–0.074	–2.151	**	–0.057	–1.664	*
Education	0.044	1.400		0.033	1.062	
Place	–0.069	–2.254	**	–0.071	–2.319	**
Independent information focus				–0.025	–0.761	
Interdependent information focus				–0.087	–2.646	***
*R2*		0.017			0.026	
*F for change in R2*		3.917	***		5.523	***
*F for Model Fit*		2.942	***		3.642	***

**C. Anxiety**	**Model 1**			**Model 2**		
	β	**T**		**β**	**T**	

Gender	–0.016	–0.522		–0.014	–0.467	
Age	–0.064	–1.933	*	–0.062	–1.842	*
Income	–0.043	–1.261		–0.039	–1.124	
Education	–0.018	–0.557		–0.021	–0.670	
Place	–0.031	–0.996		–0.029	–0.935	
Independent information focus				–0.066	–2.037	**
Interdependent information focus				0.004	0.110	
*R2*		0.008			0.012	
*F for change in R2*		1.851			2.384	*
*F for Model Fit*		1.923	*		2.063	**

**D. Anger**	**Model 1**			**Model 2**		
	**β**	**T**		**β**	**T**	

Gender	–0.004	–0.138		–0.002	–0.077	
Age	–0.011	–0.321		–0.005	–0.152	
Income	–0.046	–1.333		–0.037	–1.076	
Education	0.011	0.355		0.005	0.170	
Place	–0.076	–2.463	**	–0.076	–2.462	**
Independent information focus				–0.036	–1.112	
Interdependent information focus				–0.035	–1.045	
*R2*		0.007			0.011	
*F for change in R2*		1.648			1.963	
*F for Model Fit*		1.549			1.682	

**E. Disgust**	**Model 1**			**Model 2**		
	**β**	**T**		**β**	**T**	

Gender	0.011	0.365		0.012	0.393	
Age	–0.014	–0.414		–0.012	–0.369	
Income	–0.046	–1.331		–0.044	–1.252	
Education	0.039	1.239		0.037	1.173	
Place	–0.037	–1.207		–0.036	–1.175	
Independent information focus				–0.033	–1.013	
Interdependent information focus				0.001	0.042	
*R2*		0.005			0.006	
*F for change in R2*		1.203			0.595	
*F for Model Fit*		1.022			0.906	

*p < 0.10. **p < 0.05. ***p < 0.01.

To better illustrate the mediation role of the two coping strategies, we used the bootstrap technique. We set the number of bootstrap samples for bias-corrected bootstrap confidence intervals at 5,000 and the confidence level for all confidence intervals in output at 95% and selected the fourth model. We inputted each “negative emotion” at T_2_ as the dependent variable; “negative emotion” at T_1_ as the independent variable; and “independent information focus” and “interdependent information focus” as mediating variables. Table 4 illustrated these results. The data further confirmed the role of independent information in reducing fear [0.0112, 0.0414] (excluding zero) and anxiety [0.0157, 0.0471] (excluding zero), and the role of interdependent information in decreasing sadness [0.0120, 0.0413] (excluding zero).

**TABLE 4:** Mediation Role of independent information focus and interdependent information focus.

**Mediation path**	**Effect**	**Boot SE**	**Boot LLCI**	**Boot ULCI**
Fear T_1_ - Independent information focus - Fear T_2_	0.0232	0.0075	0.0112	0.0414
Fear T_1_ - Interdependent information focus - Fear T_2_	0.0019	0.0042	–0.0008	0.0181

Anxiety T_1_ - Independent information focus - Anxiety T_2_	0.0286	0.0078	0.0157	0.0471
Anxiety T_1_ - Interdependent information focus - Anxiety T_2_	0.0022	0.0041	–0.0050	0.0111

Sadness T_1_ - Independent information focus - Sadness T_2_	0.005	0.0053	–0.0043	0.0164
Sadness T1 - Interdependent information focus - Sadness T_2_	0.024	0.0073	0.0120	0.0413

Anger T_1_ - Independent information focus - Anger T_2_	0.0032	0.0048	–0.0061	0.0131
Anger T_1_ - Interdependent information focus - Anger T_2_	0.0026	0.0046	–0.0060	0.0125

Disgust T_1_ - Independent information focus - Disgust T_2_	0.0026	0.0039	–0.0042	0.0115
Disgust T_1_ - Interdependent information focus - Disgust T_2_	0.0036	0.0039	–0.0034	0.0124

**FIGURE 2:**
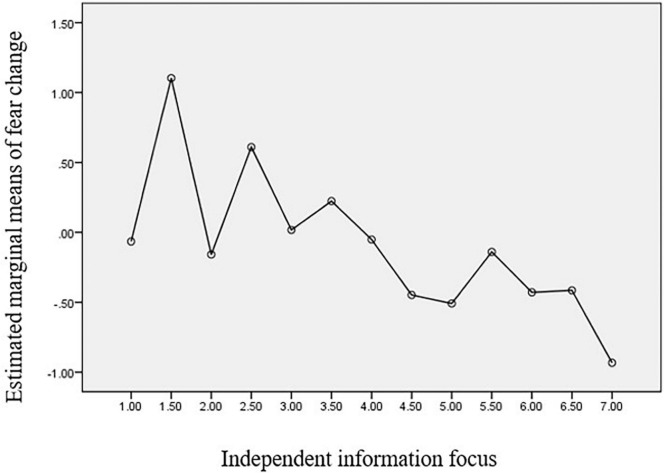
Estimated Marginal Means of Fear Change.

**FIGURE 3:**
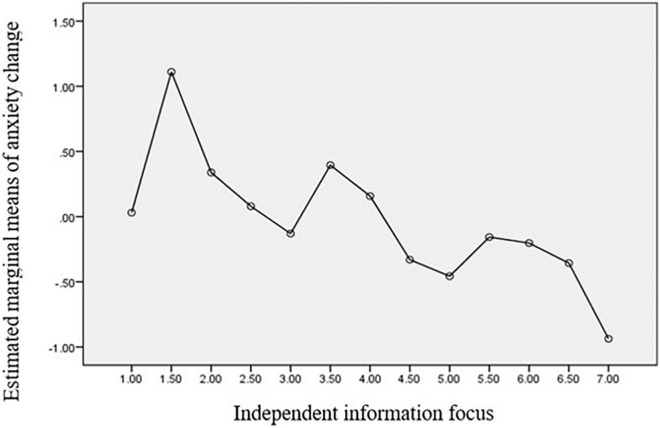
Estimated marginal means of anxiety Change.

**FIGURE 4:**
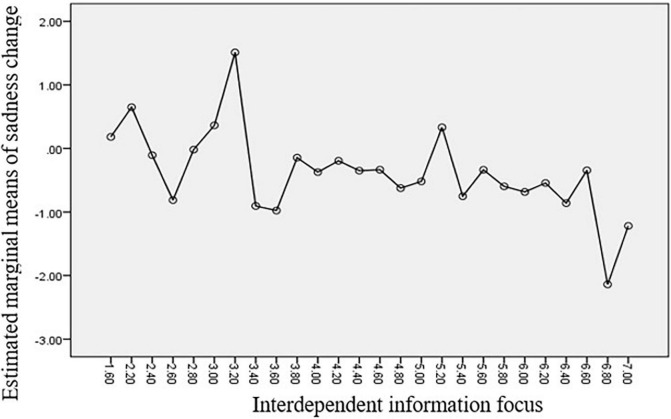
Estimated marginal means of sadness Change.

Figures 2–4 clearly show the relationship between the coping strategies and emotions in question. According to the results, independent information significantly reduces individuals’ fear and anxiety, while interdependent information contributes to relieving sadness.

## Discussion

Outbreaks of viruses are one of the most significant threats to humanity. Hence, we need to learn from the COVID-19 pandemic to prepare for future public health crises. Our research provides insight on the management of individuals’ negative emotions during crises from a cognitive perspective. We mainly discussed problem-focused coping strategies since they are the more obvious reaction to mortal threats (Han et al., 2015). Building on the theory of psychological distance and self-construal, we proposed two coping strategies (independent vs. interdependent information focus) which were effective in reducing negative emotions elicited by the COVID-19 pandemic. It is important to note that at both instances of data collection, the lockdown was still in effect in China. Yet, all negative emotions subsided in Hubei and other provinces. Addressing previous concerns about quarantine during the initial outbreak (Rubin and Wessely, 2020), the results proved that negative emotions decreased when the severity of the situation lessened, even though the lockdown measures were still in effect.

Specifically, individuals felt fear and anxiety if they could not obtain sufficient information about the virus during the pandemic. As a result, they understood the situation from an independent-self standpoint and actively searched for independent information to increase their knowledge about the virus. This, in turn, relieved their fear and anxiety. In addition, individuals experiencing sadness also sought after information pertaining to the virus to relieve their emotional state. However, independent information alone would not have a significant effect because it could not change the fact that people were suffering. However, interdependent information provided another perspective for sad individuals to find solutions in the long-term. Moreover, those experiencing anger tended to understand the virus from the interdependent-self view. They sought after information about the relationship between humans and wildlife/nature. Unfortunately, this interdependent information could not change others’ behaviors, such as eating wildlife. Hence, it could not effectively reduce anger.

Our research confirmed the existence of disgust during the COVID-19 pandemic, while demonstrating the simultaneous decreasing trends of this emotion and the decreasing severity of the outbreak. However, we failed to identify the coping approach for disgust at the cognitive level. We believe that this failure owed to the complexity of this emotion, which related more to the threat of shame. According to Han et al. (2015), coping for shame is more emotion-focused. Hence, we assumed that disgust required emotion-focused coping rather than problem-focused coping in its regulation.

We have proposed two novel concepts, independent information and interdependent information, and further improvement and testing of the constructs of these two concepts is necessary in future studies. In addition, longitudinal data, rather than laboratory data, investigated the effectiveness of these coping strategies. However, longitudinal data may include some potential confounding effects, therefore, continuous laboratory studies in the future could be beneficial in excluding confounding factors.

### Future Research

Van Bavel et al. (2020) emphasized the importance for the social and behavioral sciences to contribute to the management of the COVID-19 pandemic and its effects. Scholars have also underlined how the central recovery tasks should include a framework for coping with negative emotions (Li W. et al., 2020). Aiming to provide insights into emotion regulation during and after the outbreak, our study compared the effectiveness of coping strategies based on longitudinal data. Many scholars have already proposed the regulation of negative emotions, but there is scant evidence to support the actual effectiveness of such strategies during the COVID-19 pandemic. Hence, our research on these coping strategies, and the findings thereof could also apply to future public health crises.

While social distancing became the predominant measure to slow the spread of the virus thus far, individuals could only regulate negative emotions through limited actions. There is, therefore, an urgent need to explore coping strategies that authorities can implement flexibly and globally. Compared to behaviors, a cognitive approach is easier to guide. However, most available research has discussed coping strategies from a behavioral approach rather than a cognitive one. Some scholars have demonstrated that individuals would focus on negative (vs. positive) information when they felt certain emotions (Yiend, 2010; Tamir, 2016). However, only a few studies identified information attention as a coping strategy. Hence, to the best of our knowledge, this is the first study to discuss the cognitive coping strategies of negative emotions. Moreover, we proved the effectiveness of independent and interdependent information focus strategies in mitigating the negative emotions in question. This novel way of information classification provides insight for researchers to understand individuals’ thought processes and the kinds of information they would need during disasters.

Another limitation of previous research on emotion regulation during public emergencies was the lack of emotional distinction. The majority of studies either focused on one of the negative emotions or treated as a whole. Continuing the discussion about their differences (e.g., Raghunathan and Pham, 1999; Lerner and Keltner, 2001), our research focused on five negative emotions elicited by the COVID-19 outbreak and matched effective coping strategies to each emotion. The findings highlighted the importance of distinguishing each negative emotion and its relevant coping strategy in major public emergencies in future studies.

### Policy Suggestions

As previously mentioned, our study emphasized the significance of distinguishing negative emotions during the pandemic and confirmed that individuals sought after different information to cope with these emotions. The findings should prompt governments to attach importance to identifying the pervasive sentiment during each stage of the outbreak, in accordance with the severity of given areas, and to implement tailor-made intervention strategies to alleviate individuals’ negative emotions effectively.

In terms of communication, governments need to release relevant information based on a scientific and programmatic plan. Specifically, for individuals experiencing fear and anxiety, governments could provide the following information: (1) accurate and timely data about the current situation, such as the number of new infections and recoveries, (2) government’s efforts to control the spread of the virus, and (3) professional suggestions regarding protection, such as how to appropriate masks. For individuals experiencing sadness or anger, governments can communicate information about: (1) the role that humans play in the ecosystem, (2) the relationship between humans and wildlife, and (3) the measures taken to protect wildlife.

Moreover, we collected our second round of data when the national number of new infected cases was zero, before the government’s official termination of the lockdown measures. Despite this, all five negative emotions significantly subsided in both Hubei and other provinces. The data indicated that the severity of the situation had a higher possibility of inducing negative emotions in the public than the lockdown measures did. Hence, we highly recommend policymakers to implement measures to control the outbreak and reduce the number of new infections, rather than focusing on tightening or loosening restrictions.

## Conclusion

Building on the longitudinal data gathered during the COVID-19 outbreak, we found that individuals tended to view the pandemic from an independent-self standpoint and focus on “immediate” information related to the current status of the virus (independent information focus) if they felt fear, sadness, and anxiety. Conversely, they would view it from the interdependent-self standpoint and focus on information about the relationship between humans and nature (interdependent information focus) to cope with fear, sadness, and anger. However, independent information was only effective in decreasing fear and anxiety, while interdependent information significantly reduced sadness. We attributed this difference to the causes of each emotion. This finding could contribute to the recovery management of the COVID-19 pandemic and apply to future public emergencies.

## Data Availability Statement

The raw data supporting the conclusions of this article will be made available by the authors, without undue reservation.

## Ethics Statement

Ethical review and approval was not required for the study on human participants in accordance with the local legislation and institutional requirements. The patients/participants provided their written informed consent to participate in this study.

## Author Contributions

YS and YL performed material preparation, data collection, and analysis. YW and FL assisted in data collection and analysis. YL wrote the first draft of the manuscript. All authors commented on previous versions of the manuscript, read and approved the final manuscript, and contributed to the study conception and design.

## Conflict of Interest

The authors declare that the research was conducted in the absence of any commercial or financial relationships that could be construed as a potential conflict of interest.

## Publisher’s Note

All claims expressed in this article are solely those of the authors and do not necessarily represent those of their affiliated organizations, or those of the publisher, the editors and the reviewers. Any product that may be evaluated in this article, or claim that may be made by its manufacturer, is not guaranteed or endorsed by the publisher.
